# Topical Review: Clinical, Physiological, and Functional Benefits of Home-based Telerehabilitation with Occupational Therapists for Low Vision

**DOI:** 10.63144/ijt.2025.6703

**Published:** 2025-06-12

**Authors:** Rebecca L. Kammer, Reilly Federici, Stasi Gormley

**Affiliations:** 1 Samsara Vision, Inc., Far Hills, New Jersey, United States; 2 Seton Hall University, South Orange Village, New Jersey, United States; 3 Stasi’s Low Vision Therapy, LLC, Mount Pleasant, South Carolina, United States

**Keywords:** Age-related macular degeneration, Low vision, Low vision rehabilitation, Occupational therapy, Telerehabilitation

## Abstract

For patients with low vision, rehabilitation enables the performance of daily activities and the acquisition of skills while enhancing quality of life, despite vision loss. Access to comprehensive low vision rehabilitation services, however, is often limited. The rise of telehealth during the COVID-19 pandemic has facilitated innovative delivery of healthcare, including telerehabilitation for low vision. This literature review was undertaken to evaluate the current evidence regarding telerehabilitation conducted by occupational therapists for patients with low vision. In this review, studies investigating the effects of new programs largely found significant improvements in outcomes. Results of a multicenter, randomized controlled trial found that reading ability significantly improved and results did not differ between therapies conducted through telerehabilitation or in-office. Additionally, studies surveying providers and patients regarding their sentiments about telehealth found that comfort level and overall satisfaction were similar between in-office visits and telerehabilitation.

Individuals seeking rehabilitation services for low vision face a complex array of challenges and have a highly particular set of needs. More than 3 million people in the United States (US) had low vision in 2020, and by 2050, it is estimated that this number will reach almost 7 million (Jackson et al., 2023). Approximately 73% of patients with low vision in the US are 65 years of age and above, and more than 50% of patients with vision loss have other vision- or age-related comorbid conditions, such as mood disorders, which contribute to lower overall functioning and health. Patients with low vision are often at heightened risk for falls and may have difficulties reading and managing medications (Goldstein et al., 2012; Riddering, 2016). Vision-related transportation barriers, including the inability to safely drive, are also common (Goldstein et al., 2012; Klauke et al., 2023; Riddering, 2016). Loss of vision may also cause waning interest and ability to participate in activities that give life purpose, security, and pleasure (Klauke et al., 2023).

The COVID-19 pandemic had a galvanizing effect on technological innovation in new models of remote healthcare delivery, and some models pioneered during this period have proven beneficial compared with traditional models (McAlearney et al., 2022; Shaver, 2022). In-home telerehabilitation services for patients with low vision represent a patient-centric remote-care model that has the potential to improve outcomes by eliminating the need for travel, improving convenience and engagement, and harnessing emotional and psychological connections to the home that can improve home-based tasks (Bittner et al., 2023).

Vision telehealth and remote training reports are often provided by low vision therapists or optometrists. Remote care provided by occupational therapists (OTs) may have distinct advantages for patients with low vision, as there are many complex concerns, such as psychosocial impact, fall risk, and instrumental daily activities, that OTs are uniquely equipped to address.

The American Journal of Occupational Therapy 2020 Practice Guideline examined three systematic reviews, including 38 total articles, and concluded that there is strong evidence supporting the role of OTs for older adults with low vision. The guideline recommends the routine use of low vision rehabilitation for activities of daily living (ADL) (Kaldenberg & Smallfield, 2020). Unfortunately, the number of practicing OTs trained in low vision rehabilitation appears insufficient to meet the immense and growing need for their expertise. It is estimated that for every 40,000 patients with low vision in the US, there is only one low vision-certified OT. The number of enrolling students and recent graduates from OT master’s and doctoral programs is also low relative to the projected need (Harvison, 2022; Weisser-Pike et al., 2023).

Telerehabilitation may mitigate this growing gap. In a recent scoping review of the literature, low vision telerehabilitation was shown to be effective in improving self-reported visual outcomes and quality of life and was generally well accepted by patients (Jones et al, 2022). The study cited several key advantages of telerehabilitation over traditional models, including the potential to reduce costs, extend provider reach, and surmount geographic and travel barriers. However, the study did not specify whether OTs or another kind of therapist provided care in each described study. With this background, we performed a literature review to quantify and describe the current evidence regarding the use of OT-delivered telerehabilitation for patients with low vision.

## Methods

To assess the current landscape of low vision telerehabilitation provided by OTs, we conducted a literature review across two major databases (*PubMed* and *Google Scholar*) to identify studies that investigated approaches to telerehabilitation in patients with low vision that included OTs as either providers or participants. Articles were not excluded if they also included other kinds of providers in addition to OTs. The keywords used in the search included (“telerehab*” OR “telemed*” OR “telehealth”) AND (“low vision” OR “age-related macular degeneration” OR “glaucoma” OR “visual impairment”) AND (“occupational therap*”). Abstracts identified by the search strings were then screened for inclusion. The search was limited to original research written in English and published between January 2012 and May 2024; meta-analyses and commentaries/editorials identified by the search strings were excluded.

## Results

### Study Characteristics

Our search identified seven articles that met the criteria for inclusion in this review. Identified articles were analyzed and grouped based on the content of the study, as determined by author review: four articles investigated the results of a new program for telerehabilitation of patients with low vision that included OTs, which may have included other providers as well (*Investigations of New Programs*); two articles surveyed patients and providers, including OTs, to gain an understanding of how they felt about the use of telerehabilitation for addressing visual loss (*Investigations of Patient Sentiment*); and one article was a multicenter, randomized controlled trial investigating the effects of telerehabilitation on the reading ability of patients with low vision, which included OTs along with other providers (*Investigation of Telerehabilitation vs In-Office Training)*. Brief summaries of the included studies are available in Table 1 (Aravich & Stants, 2022; Bittner, Kaminski, et al. 2022; Bittner et al, 2024; Bittner, Yoshinaga, et al. 2022; Dunne et al., 2020; Kaldenberg & Smallfield, 2017; Tinelli et al., 2017).

### Investigations of New Programs

Kaldenberg and Smallfield (2017) were among the first to challenge the traditional reliance on in-person care by investigating whether legally blind individuals could learn to use a digital tablet to perform telerehabilitation tasks at home. In their feasibility study, researchers recruited four patients (mean age: 74 years; VA range: 20/160 to 20/4000) who had never used a tablet to participate in ten 90-minute weekly group sessions to learn how to use a tablet as a low vision assistance device for home use. Each week, participants were taught a new skill or app, including basic tools (e.g., camera, mail, contacts, clock, calendar, web browser, and video calling) and low vision–specific items (e.g., color identification, magnification, voice recorder, and medication management). The classes were conducted in person by OT graduate students who were supervised by the studies’ Primary Investigator, and participants then carried out the instructed telerehabilitation practices at home asynchronously following training sessions. At week 10, significant changes were detected, with Canadian Occupational Performance Measure scores improving by 3.45 and 3.65 for mean total performance and satisfaction, respectively. At the 3-month telephone follow-up visit, the frequency of tablet usage remained significantly higher compared to baseline.

In another pilot study (Tinelli et al., 2017), a team of researchers in Italy developed and tested a novel in-home audiovisual telerehabilitation (AVT) system for the treatment of hemianopia secondary to stroke- or neurosurgery-induced chronic brain lesions. The AVT system consisted of a portable tabletop apparatus that delivered visual and acoustic stimuli over a 180° frontal range. During asynchronous rehabilitation sessions, patients performed trainings and evaluations consisting of predefined combinations of visual and acoustic stimulation, which were programmed and reviewed by a remote therapist.

During the rehabilitation sessions, three types of stimuli were delivered: (1) unimodal visual stimuli (presentation of only visual stimuli); (2) unimodal acoustic stimuli (presentation of only acoustic stimuli); and (3) bimodal audiovisual stimuli (visual stimuli accompanied by sounds). Patients were asked to look at a central fixation point and then shift their gaze toward the visual stimulus without head movement. When the patient saw the visual target, they indicated this by pressing a response button; the presentation of the stimuli only occurred if the subject was looking at the central fixation point, as detected by the camera. Furthermore, the intensity and type of stimulation of the visual field varied according to the site of the visual field defect.

The system was tested in prospective case studies among three adults with hemianopia secondary to iatrogenic or post-stroke brain lesions. All three participants complied with the 5-times-per-week training regimen over approximately 5 weeks and found the setup flexible and easy to use at home, and all participants showed improvements in visual detection abilities in training-specific tasks even after a period of 6 to 12 months following the cessation of training. The study concluded that the telerehabilitation system provided advantages over in-clinic or home-visit formats, including empowering patient autonomy and improving access (Tinelli et al., 2017).

Bittner and colleagues (Bittner, Kaminski, et al., 2022) expanded a previously examined telerehabilitation model in a prospective pilot study to include OTs in collaboration with optometrists who prescribed magnifying devices from multiple centers. After baseline evaluation, 14 patients with low vision (mean age, 68 years) received a series of two in-home individualized training sessions via Zoom with a vision-rehabilitation provider (four optometrists and one OT provided the trainings) located remotely. The mean time between sessions was 39 days (range: 21–79 days; SD: 16 days). Following the first training, significant improvements were observed in mean reading acuity and mean reading speed relative to baseline.

Another study (Aravich & Stants, 2022) examined the results of a hybrid in-office/telerehabilitation pilot program implemented by the University of Pittsburgh Medical Center Low Vision Occupational Therapy Department, in which 15 patients aged 19 to 95 years with a variety of low vision diagnoses were provided telehealth services and access to a supplemental device lending library. Patients enrolled in this program demonstrated a decrease in missed appointments; notably, participants in the hybrid program missed less than 1% of their appointments, whereas patients with low vision have been reported as missing in-clinic appointments at rates up to 50%. When patients were asked (on a 5-point Likert scale) if they agreed that the hybrid program was beneficial, seven patients strongly agreed, five agreed, and three neither agreed nor disagreed. The number of patients who found telehealth services to be helpful increased during the course of the program. Participants also noted that the hybrid program granted them increased independence with daily activities, such as meal preparation, medication management, reading, leisure, technology use, and money management.

### Investigations of Patient Sentiments

Dunne and colleagues (2020) performed focus groups and interviews with 32 patients who had visual loss following a stroke. As part of the study, they also interviewed 10 of the patients’ care providers and 24 OTs to identify facilitations and barriers to using rehabilitation tools and to identify elements of good practice in telerehabilitation. The care providers and OTs specifically evaluated the Durham Reading and Exploration (DREX) training app. A total of five interviews were conducted and included five stroke survivors and four care providers. The remaining stroke survivors (n=27), care providers (n=6), and OTs (n=24) were not interviewed individually but were included in focus groups. Separate focus groups were carried out for the OTs and for the patients/care providers. The focus groups that included OTs focused more on understanding the facilitations and barriers that they experience in supporting stroke survivors and their perspectives on the use of technology in post-stroke visual rehabilitation. The focus groups and interviews were semi-structured, with six open-ended questions used to prompt discussion and follow-up questions that depended on responses. Focus groups were led by the primary author of the study and were carried out across five different venues in the United Kingdom, whereas the interviews were conducted in patients’ homes. Each focus group and interview lasted about 60 minutes.

In this study, Dunne and colleagues identified two major themes as barriers to the use of telerehabilitation in stroke care: acute ward rehabilitation and the perceived disadvantages of technology. They also identified one major theme that represents a facilitation to telerehabilitation in stroke care: that technology and the internet are perceived as advantageous. OTs noted that the limited time they get to spend with stroke survivors in acute settings was a barrier to their rehabilitation efforts and were optimistic about the prospect of a form of therapy, such as telerehabilitation, that would enable them to train a patient who could then employ the therapy on their own time, without direct therapist supervision (patients are still monitored remotely at various time points). Both OTs and patients expressed ambivalent views toward technology in telerehabilitation for stroke care. OTs reported that they felt as though it was too much of a “jump” for patients to adopt new technology after being discharged from an acute care ward. While many patients were frightened by the prospect of having to use or learn a new technology during rehabilitation and the potential isolation caused by a lack of in-person contact, others noted that the repeatability of online tasks and videos made them more accessible. The ability to repeat therapeutic tasks was identified as a necessary aspect of rehabilitation by some patients (Dunne et al., 2020).

In another study (Bittner, Yoshinaga, et al., 2022), a satisfaction survey was distributed to 58 patients with visual impairment who were enrolled in studies involving telerehabilitation conducted in three phases spanning a 7-year period. Phase 1 was a prospective cohort study that connected participants with low vision to telerehabilitation via videoconferencing in their homes and was conducted from 2016 to 2017 (n=10). Phase 2 was similar to phase 1, yet community Lions Club members assisted participants to set up the telerehabilitation sessions in the participants’ homes and was conducted from 2018 to 2019 (n=11). Phase 3 was a randomized controlled trial in which participants were allocated to either telerehabilitation from home or in-office rehabilitation and was conducted from 2020 to 2022 (n=24). Across all phases, telerehabilitation was provided by either optometrists (at seven sites) or OTs (at three sites).

Each participant was involved in only a single phase, and therefore, each participant represented a unique telerehabilitation encounter that was not captured in another phase. However, participants in all three study phases completed the same satisfaction survey. Results from these multiphase and multicenter studies indicated that using telerehabilitation for remote training with optical or electronic magnifiers had high levels of acceptability among visually impaired individuals. No significant differences were revealed in the participants’ comfort level, overall satisfaction, self-rated improvement in magnifier use after the session, or interest in having another session in the future between in-office trainings and telerehabilitation sessions. Across all three phases, ratings for being very satisfied with telerehabilitation were associated with the belief that videoconferencing was as accurate as in-person care. Participants with portable electronic video magnifiers were more likely to indicate improvement post-telerehabilitation than those with optical magnifiers.

### Investigation of Telerehabilitation vs In-Office Training

Expanding upon their previous work, Bittner and colleagues (2024) performed a multicenter, randomized controlled trial to determine the differences between outcomes for telerehabilitation or in-office training with magnification devices for low vision. In this study, 61 patients with visual impairment were randomized to telerehabilitation or in-office training at either an academic center or a private practice 1 to 4 months after receiving new portable electronic, hand-held, or stand optical magnifiers. Vision rehabilitation, both via telerehabilitation and in-office care, was provided by one of either eight optometrists or two OTs. Patients randomized to telerehabilitation received a kit with loaner equipment to be used instead of their own internet-enabled devices. While differing types of internet-enabled loaner devices were provided to patients randomized to telerehabilitation, all patients received the same stand for the device and the same standardized near acuity cards to assess reading with the magnifier.

Videoconferencing for patients randomized to telerehabilitation was performed via Zoom, and sessions lasted approximately 1 hour. Among the 47 patients who completed the trial, reading ability with new magnifiers improved significantly by 0.61 logits on average (95% confidence interval [CI], 0.36–0.86; P<0.001), and results were similar for telerehabilitation (mean improvement=0.44 logits; 95% CI, 0.08–0.80; P=0.018) and in-office trainings (mean improvement=0.43 logits; 95% CI, 0.15–0.71; P=0.003). There were no significant differences in follow-up visits between randomized groups. The authors found that using telerehabilitation to conduct follow-ups for newly prescribed magnifiers or other reading aids can offer a convenient, safe, and resource-efficient means for rendering vision rehabilitation services, and suggested that, based on these data, a larger-scale noninferiority trial be carried out to determine if telerehabilitation is at least as effective as in-office care (Bittner et al., 2024).

## Discussion

The home takes on an increasingly central role with age and has physical, behavioral, cognitive, emotional, and social significance (Hatcher et al., 2019). Aging adult populations have consistently shown a strong preference for residence at home rather than in nursing homes, reflected in notably higher quality of life scores among those living in their homes (Hedayati et al., 2014; Kok et al., 2015). For adults experiencing low vision, active engagement in household activities is crucial for achieving independent living and overall well-being (Stevens-Ratchford & Krause, 2004). Additionally, as noted by Aravich and Stants (2022), older adults are more likely to have multiple comorbidities and be at heightened risk of complications, which further highlights the benefits of a care delivery model that enables patients to receive rehabilitation services from home without being exposed to the potential risks of an acute-care environment. As the one multicenter, randomized controlled trial identified in this review (Bittner et al., 2024) found equally efficacious outcomes between telerehabilitation and in-office visits for low-vision rehabilitation. Further efforts to develop telerehabilitation programs for low vision may reflect an important opportunity to maximize the availability of care without sacrificing efficacy.

Although older adults have reported high levels of receptibility to and acceptance of the use of telehealth services, their engagement with telehealth remains lower than other age groups, which can largely be attributed to technology-enabling factors such as internet-connected device ownership and operating knowledge of such devices (Choi et al., 2022). Therefore, studies that connect older adults to new technologies and provide them with training on how to use these technologies, such as those identified by this review (Aravich & Stants, 2022; Bittner, Kaminski, et al., 2022; Kaldenberg & Smallfield, 2017; Tinelli et al., 2017), may prove especially helpful in bridging this gap.

Looking ahead, an aging population will place increased strain on the healthcare system, and the demand for ophthalmic services can be expected to increase (Patel et al., 2021). Telerehabilitation is poised to bridge a growing divide in the delivery of low vision services to aging patients by improving access and convenience, overcoming geographical constraints and transportation barriers, and leveraging psychological benefits of in-home care. The home environment provides a sense of normalcy and familiarity, potentially reducing stress and promoting a positive experience that is conducive to learning. The positive reinforcement and encouragement associated with telerehabilitation may help patients with low vision nurture self-belief and stay motivated and committed to their rehabilitation.

In our review, the identified studies supported the use of low vision telerehabilitation as both effective and acceptable to patients. Although this review did not identify any research comparing outcomes from low vision telerehabilitation services as provided by OTs vs other low vision care providers, such as optometrists or low vision therapists with different training, the results of the identified studies showed that OTs can provide low vision telerehabilitation services in an efficacious manner that is acceptable to patients. This is consistent with both prior research on in-person programs, which have demonstrated that low vision rehabilitation offered by OTs can be an effective way to promote independence and increase reading ability in older adults, and with the most current version of the American Academy of Ophthalmology’s Vision Rehabilitation Preferred Practice Pattern, which supports the use of an OT for many aspects of vision rehabilitation (Jackson et al., 2023; Liu & Chang, 2020; Smallfield et al., 2013).

## Conclusions

Studies investigating the effects of new programs largely found significant improvements in outcomes, and results of a multicenter, randomized controlled trial found that not only did reading ability significantly improve but results did not differ between therapies conducted through telerehabilitation or at an in-office visit. Additionally, studies surveying providers and patients regarding their sentiments about telehealth found that comfort level and overall satisfaction were similar between in-office visits and telerehabilitation. While the results of these studies are very promising, the relative dearth of publications regarding the use of telerehabilitation by OTs for patients with low vision clearly demonstrates the need for greater research in this area. Research exploring differences in the effects of low vision rehabilitation services when offered by different providers, including OTs, may help characterize ideal roles and workflows.

## Figures and Tables

**Table 1:** Studies Investigating Telerehabilitation for Patients with Low Vision That Included Occupational Therapists

Study, setting, sample size	Purpose	Study design	Summary of Intervention	Data/tools	Relevant Findings
Aravich & Stants, 2022, USA, *n=*15 [adults with low vision]	*Investigations of New Programs*	Qualitative, one group, prospective pilot study	Participants were enrolled in a hybrid low vision OT program, in which they were provided with an iPad Pro pre-loaded with a health care system app, which they were trained on by an OT, and given access to a lending-library of additional low-vision assistive technology devices. Patients completed telehealth sessions with an OT in conjunction with in-clinic care as needed. OTs provided all trainings and sessions. Synchronous hybrid practice	Patient satisfaction, via both 5-point Likert scale and open-ended questions Number of sessions attended	Participants increased the frequency of OT visits, allowing for greater carryover of skills to the home environment and real-life practice. Patients reported an increase in independence with daily activities and reduced travel time and associated cost.
Bittner, Kaminski, et al., 2022, USA, *n=*14 [non-cognitively impaired, English-speaking adults who had newly received portable electron video magnifiers, hand-held or stand optical magnifiers]	*Investigations of New Programs*	Quantitative, quasi-experimental, one group, pretest–posttest research design	Participants were provided with a smartphone to use for telerehabilitation specifically and were guided and assessed by a visual rehabilitation provider over two sessions on how to use an electronic magnifier via a standardized protocol. An OT was one of the five visual rehabilitation providers who trained and assessed participants. Synchronous telerehab home practice	Best corrected visual acuity (at near via Lighthouse continuous text near reading card or MNRead test, at distance via Early Treatment of Diabetic Retinopathy Study chart or Snellen chart) Contrast sensitivity, via Peli-Robson or MARS chart Reading speed, via MNRead test	Reading speed with the magnifier improved significantly by an average of 15 words per minute for the same text size from session 1 to 2 Significant improvement in reading speed by 0.18 log words per minute for the same text size
Bittner, Kaminski, et al., 2024, USA, *n=*47 [visually impaired adults]	*Investigation of Telerehabilitation vs In-Office Training*	Prospective, quantitative multicenter, randomized controlled trial	Low vision patients were randomized to receive either telerehabilitation (*n=*29) or in-office training (*n=18)* on how to use new portable, hand-held, or stand optical magnifiers. Telerehabilitation included loaner equipment for Zoom videoconferencing with remote control access software. OTs were two of the 10 providers conducting the telerehabilitation/in-office trainings. Synchronous telerehabilitation	Proportion of telerehabilitation participants with observed errors, via provider observation Reading ability, via Activity Inventory Telephone Interview for Cognitive Status (TICS) Hospital Anxiety and Depression Scale	Reading ability improved after magnifiers were provided, with further improvements following additional magnifier training via either telerehabilitation or in-office usual care, with no difference between telerehabilitation and in-office interventions
Bittner, Yoshinaga, et al., 2022, USA, *n=*45 [participants in 3 studies on telerehabilitation for low vision patients using magnification devices]	*Investigations of Patient Sentiment*	Multicenter, quantitative, retrospective analysis of survey responses and visual acuity from participants across 3 studies	Participants in 3 studies on telerehabilitation for low vision (phase 1, phase 2, and phase 3) were all given the same satisfaction survey. OTs served as 3 of the 10 providers performing the vision rehabilitation trainings	Patient sentiment regarding telerehabilitation, via survey Visual function with or without a magnifier, via distance and near best corrected visual acuity	The majority of patients (68%) across all phases and groups agreed that telerehabilitation was as accurate as in-person trainings, with 83% of patients being somewhat or very interested in receiving training again via the same modality. No patients in any phase or group reported not being satisfied with telerehabilitation Patients reported similar visual function outcomes regardless of if they received in-office or telerehabilitation
Dunne et al., 2020, UK, [*n=*66, 34 stroke survivors with partial vision loss, 10 carers, and 24 OTs]	*Investigations of Patient Sentiment*	Prospective, qualitative semi-structured interviews	Survey data informed the creation of 12 interviews (*n*=5) and focus groups (*n*=7) with stroke survivors with visual loss, carers for stroke survivors, and OTs, as part of a subset of a larger study investigating barriers and facilitators in stroke rehabilitation. Each session lasted roughly 60 minutes. OTs served as respondents in two focus groups.	Patient sentiment regarding barriers and facilitators to stroke rehabilitation, collected verbally with focus groups and analyzed using thematic analysis	Identified barriers included lack of confidence with technology, the perceived fear of using telerehabilitation, and the reduced face-to-face contact associated with technological solutions in rehabilitation. Recommendations moving forward were to include better education for patients about the requirements of training packages, additional goal setting, the need for repetition, the need for feedback mechanisms, and use of a multimodal approach (e.g., paper, text, audio, visual elements, etc.) that enables these resources to be understood and used by all stakeholder populations
Kaldenburg & Smallfield, 2017, USA, *n*=4 [older women]	*Investigations of New Programs*	Quasi-experimental, quantitative, one-group, pretest–posttest research design with a 3-month telephone follow-up	10 weekly in-person group sessions, each lasting approximately 90 minutes and led by a minimum of two OT graduate student researchers with supervision from the PI Asynchronous telerehab home practice	Canadian Occupational Performance Measure [COPM] Daily tablet use-minutes/hours	Improvement in both performance and satisfaction was greater than 2 points, showing meaningful clinical change as identified by previous COPM research Daily tablet use significantly increased from 15 minutes at pretest to 3 hours at posttest to 4.5 hours at follow-up.
Tinelli et al., 2017, Italy, *n=*3 [adult men with hemianopia]	*Investigations of New Programs*	Quasi-experimental, quantitative, one-group, pretest–posttest research design and follow up at months 6, 9, and 12 for subjects 1, 3, and 2, respectively	Following an initial session, trainings were self-conducted for at least 5 days a week for a period of approximately 5 weeks, with individual training times varying based on patient fatigability. An OT provided training and assessment Asynchronous telerehab home practice	Unimodal visual test Bimodal audiovisual test Computerized visual field perimetry	Significant improvement in visual detection rates in the affected hemifield in the Fixed-Eye condition in one patient and significant difference in visual detection rates in Eye-movement condition in all patients.
